# Limited Growth Recovery after Drought-Induced Forest Dieback in Very Defoliated Trees of Two Pine Species

**DOI:** 10.3389/fpls.2016.00418

**Published:** 2016-04-01

**Authors:** Guillermo Guada, J. Julio Camarero, Raúl Sánchez-Salguero, Rafael M. Navarro Cerrillo

**Affiliations:** ^1^Departamento de Botánica, Universidade de Santiago de CompostelaLugo, Spain; ^2^Instituto Pirenaico de Ecología (IPE-CSIC)Zaragoza, Spain; ^3^Departamento de Sistemas Físicos, Químicos y Naturales, Universidad Pablo de OlavideSevilla, Spain; ^4^Departamento de Ingeniería Forestal, Universidad de CórdobaCórdoba, Spain

**Keywords:** dendroecology, die-off, extreme climate event, forest resilience, *Pinus nigra*, *Pinus sylvestris*, xylem, xylogenesis

## Abstract

Mediterranean pine forests display high resilience after extreme climatic events such as severe droughts. However, recent dry spells causing growth decline and triggering forest dieback challenge the capacity of some forests to recover following major disturbances. To describe how resilient the responses of forests to drought can be, we quantified growth dynamics in plantations of two pine species (Scots pine, black pine) located in south-eastern Spain and showing drought-triggered dieback. Radial growth was characterized at inter- (tree-ring width) and intra-annual (xylogenesis) scales in three defoliation levels. It was assumed that the higher defoliation the more negative the impact of drought on tree growth. Tree-ring width chronologies were built and xylogenesis was characterized 3 years after the last severe drought occurred. Annual growth data and the number of tracheids produced in different stages of xylem formation were related to climate data at several time scales. Drought negatively impacted growth of the most defoliated trees in both pine species. In Scots pine, xylem formation started earlier in the non-defoliated than in the most defoliated trees. Defoliated trees presented the shortest duration of the radial-enlargement phase in both species. On average the most defoliated trees formed 60% of the number of mature tracheids formed by the non-defoliated trees in both species. Since radial enlargement is the xylogenesis phase most tightly related to final growth, this explains why the most defoliated trees grew the least due to their altered xylogenesis phases. Our findings indicate a very limited resilience capacity of drought-defoliated Scots and black pines. Moreover, droughts produce legacy effects on xylogenesis of highly defoliated trees which could not recover previous growth rates and are thus more prone to die.

## Introduction

Mediterranean forests are able to recover following major disturbances such as droughts by displaying high resilience (e.g., Lloret et al., 2004). However, climate warming is expected to magnify drought stress in the Mediterranean Basin by rising air temperatures and evapotranspiration rates thus amplifying drying trends (Cook et al., 2014). Warmer temperatures, when superimposed on episodes of scarce precipitation, result in severe water deficits intensifying drought impact and reducing forest growth and productivity (Williams et al., 2013). Consequently, warmer and drier conditions could lead to growth decline of drought-prone Mediterranean conifer forests (Sarris et al., 2007; Sánchez-Salguero et al., 2012a; Galván et al., 2014). Such reductions in productivity may predispose trees to drought-induced dieback once growth decline and vigor loss become irreversible (Camarero et al., 2015). Thus, it is compelling to determine if Mediterranean forest growth recovers after successive or severe droughts and if this response may cause a loss of resilience.

Severe droughts are extreme climatic events and therefore they constitute rare and unpredictable drivers of forest dynamics (Gutschick and Bassirirad, 2003). However, to account fully for droughts effects on forest growth, their extremity must be documented not only from the climatic perspective but also from the tree response (Smith, 2011). Dendrochronology facilitates the assessment of drought impacts on radial growth by reconstructing tree-ring variables since the unpredictability of droughts makes their continuous surveillance challenging (Dobbertin, 2005; Eilmann et al., 2013). Growth decline and dieback represent long-lasting impacts of severe droughts on forest productivity (McDowell et al., 2008). Tree-ring width records usually reflect a growth reduction in response to prolonged droughts before crown decline symptoms (needle loss and yellowing) appear (Torelli et al., 1986, 1999; Pedersen, 1998; Bigler et al., 2006). Frequently, conifers also show a high growth responsiveness to water availability previous to drought-triggered needle loss or tree death (Ogle et al., 2000). In addition, summer drought is also associated with more conspicuous symptoms of vigor loss as accelerated defoliation (Solberg, 2004). Furthermore, droughts cause legacy effects on tree growth thus compromising forest resilience (Anderegg et al., 2015).

Multiple dieback episodes in Mediterranean conifer forests subjected to long dry spells confirm that pine species are particularly prone to drought-induced growth decline, needle loss or defoliation and mortality (Martínez-Vilalta and Piñol, 2002; Sarris et al., 2007, 2011; Sánchez-Salguero et al., 2012a; Camarero et al., 2015). The vulnerability of some Mediterranean pines to drought stress can be explained because they are tall species (compared with co-occurring shrubby or small conifers such as junipers), display high leaf areas, show isohydric behavior characterized by a rapid stomatal closure in response to drought and present a high xylem vulnerability to embolism (McDowell and Allen, 2015). In fact, some strictly Mediterranean pine species (e.g., *Pinus halepensis*) apparently well adapted to withstand drought stress (Klein et al., 2011) can show dieback under extremely dry and warm conditions (Camarero et al., 2015; Dorman et al., 2015). This raises the question on how resilient will be pine species from different biogeographical origins to drought-induced dieback.

Here, we compare the post-drought growth responses of two pine species with Eurosiberian (Scots pine) and Mediterranean (black pine) distributions to provide a measure of resilience in similar drought-prone forests. We capitalize on a drought-induced dieback caused by severe late-20th century droughts affecting Spain (1994–1995, 1999, 2005) and leading to growth decline and enhanced defoliation in pine plantations located in SE Spain (Sánchez-Salguero et al., 2012a,b). We quantify the post-drought growth responses at inter- (tree-ring width) and intra-annual (xylem development or xylogenesis) scales in three defoliation classes since needle loss is a proxy of post-drought changes in tree vigor (cf. Dobbertin, 2005). Our specific aims are: (i) to quantify the post-dieback growth trends; (ii) to characterize xylogenesis; and (iii) to examine climate-growth associations. We explicitly fulfill these objectives by comparing three defoliation classes of the two pine species. We hypothesize that the most defoliated trees will show the lowest growth rates and the highest sensitivity to water availability, i.e., the lowest post-drought resilience capacity. It is also expected to detect this pattern more clearly in Scots pine than black pine since the former species is more vulnerable to drought-induced xylem embolism (Martínez-Vilalta et al., 2004).

## Materials and methods

### Study area and tree species

The study area is located in the Sierra de Filabres, Andalusia, SE Spain (37° 22′ N, 2° 50′ W; see Figure S1). This area was planted with Scots pine (*Pinus sylvestris* L.) and black pine (*Pinus nigra* Arn.) in the 1970s, with most stands of each species located at approximate elevations of 1850–2000 m and 1700–1850 m a.s.l., respectively (Sánchez-Salguero et al., 2012b; Herrero et al., 2013). Both Scots pine and black pine stands are among the southernmost planted forests of both species (Figure S1). Therefore, these populations can be considered marginal from both biogeographical (southernmost stands) and climatic (xeric limit) points of view, particularly in the case of Scots pine (Barbéro et al., 1998). The climate is Mediterranean of semi-arid type since the mean annual temperature is 13.4°C and the annual rainfall ranges between 350 and 450 mm. These data are based on a regional climate series calculated for the period 1970–2008 using daily and monthly climate data (mean maximum and minimum temperature, precipitation) obtained from several local stations (see Figure S2 and Table S1). For these stations we also estimated the potential evapotranspiration (PET) using values of mean temperature and solar radiation (Hargreaves, 1983). Then, we calculated the daily water balance as the difference between precipitation and PET. To characterize drought severity in the study area since the 1970s we obtained monthly values of the Standardised Precipitation-Evapotranspiration Index (SPEI) calculated for 3-, 6-, and 12-month long scales since these are the most important scales for the study pine species (Pasho et al., 2012). Negative and positive SPEI values indicate dry and wet conditions, respectively. The SPEI was calculated for the 0.5° grid including the study sites and it was obtained from the webpage http://sac.csic.es/spei/index.html. The topography of the study sites is characterized by steep slopes (>35%). Geological substrates are Paleozoic schist and quartzites leading to regosols soil types.

The study trees are located in plantations managed through selective thinning, which involves harvesting the dominated trees while retaining those within specified size classes for future natural seeding. The current density was similar across the study area, with a mean basal area of 25 m^2^ ha^−1^. More details on the study sites are available in Sánchez-Salguero et al. (2012a,b).

### Field sampling and tree selection

A stratified sampling was followed to select trees showing contrasting defoliation after the severe 2005 drought. Firstly, a systematic forest inventory was performed to select stands whose trees shared similar site conditions (soil, topography) but presented contrasting defoliation degrees (Sánchez-Salguero et al., 2012b). Secondly, 30 trees were selected for each species (10 trees per defoliation class) based on their similar size (dbh, diameter measured at 1.3 m, tree and crown heights) and age (mean ± SE = 32 ± 3 years; see also Table 1). For each sampled tree, the proportion of crown cover was estimated to the nearest 5% by comparing every tree with a reference tree with the maximum amount of foliage at each site (Schomaker et al., 2007). Finally, the trees were classified in three defoliation classes: defoliation ≤ 25% of the crown (scarcely or not defoliated trees, henceforth abbreviated as N trees), 25 < defoliation < 75% (trees with intermediate defoliation level, henceforth abbreviated as I trees), and defoliation ≥ 75% (highly defoliated trees, henceforth abbreviated as D trees).

**Table 1:** **Main characteristics of the study trees**.

**Pine species**	**Defoliation class (code)**	**No. trees**	**Defoliation (%)**	**Dbh, diameter at 1.3 m (cm)**	**Height (m)**	**Crown height (m)**
Scots pine *(Pinus sylvestris)*	Severe defoliation (D)	10	84.0 ± 1.9c	16.1 ± 0.2	6.2 ± 0.5	1.9 ± 0.7
	Intermediate defoliation (I)	10	40.0 ± 2.7b	15.7 ± 1.5	6.5 ± 0.5	2.3 ± 0.6
	Scarce or no defoliation (N)	10	11.0 ± 1.9a	16.8 ± 0.9	7.1 ± 0.5	2.5 ± 0.5
Black pine *(Pinus nigra)*	Severe defoliation (D)	10	85.0 ± 3.5c	14.4 ± 1.0	6.7 ± 0.7	1.9 ± 0.6
	Intermediate defoliation (I)	10	50.0 ± 3.5b	15.6 ± 0.8	7.2 ± 0.5	2.3 ± 0.8
	Scarce or no defoliation (N)	10	12.0 ± 2.0a	16.9 ± 0.5	7.4 ± 0.4	1.8 ± 0.4

*Values are means ± SE. Different letters show significant differences (P < 0.05) between defoliation classes within each species according to S-N-K post-hoc tests*.

### Dendrochronological sampling and processing

Dendrochronological sampling was carried out in winter 2008. Two cores per tree at breast height were collected at 1.3 m from 30 trees per species by using a Pressler increment borer. The cores were air dried, stuck onto wood guides with glue, and sanded using progressively finer grain papers until the rings were clearly distinct (Fritts, 2001). Then, tree rings were visually cross-dated and measured with a resolution of 0.01 mm using a semi-automatic LINTAB device (F. Rinn, Heidelberg, Germany). Cross-dating was checked using the COFECHA software (Holmes, 1983).

Since basal-area increment (BAI, cm^2^ year^−1^) is assumed to be a meaningful indicator of tree growth because it removes variation in growth attributable to increasing circumference and it is related to the transpiring crown surface (e.g., Linares et al., 2009), we converted tree-ring widths into BAI. We assumed a circular shape of stem cross-sections and used the following formula:

(1) $$\begin{array}{l} \text{BAI}=\pi \left({\text{R}}_{t}^{2}-{\text{R}}_{t-1}^{2}\right) \end{array}$$

where *R* is the radius of the tree and *t* is the year of tree ring formation. Mean series of BAI were obtained for the two species and the three defoliation classes.

To calculate climate-growth relationship at inter-annual scales we transformed tree-ring widths into indices following standard dendrochronological procedures (Fritts, 2001). The individual tree-ring width series were double-detrended using negative linear or exponential functions and cubic smoothing splines with a 50% frequency-response cut-off at 20 years to preserve high-frequency variability. Observed width values were divided by fitted values to obtain ring-width indices. Autoregressive modeling was performed on each detrended ring-width series to remove part of the first-order autocorrelation. Then, these indices were averaged using a biweight robust mean to obtain residual chronologies for each species and defoliation class. All chronologies were built using the program ARSTAN (Cook and Krusic, 2005). Finally, we calculated two dendrochronological statistics (first-order autocorrelation, mean sensitivity) and compared their mean values between defoliation classes to assess growth patterns.

### Xylogenesis

Tree rings are produced by the cambium which generates tracheids differentiating through developmental stages (radial enlargement, wall thickening) until becoming mature (Mahmood, 1971; Wodzicki, 1971, 2001; Larson, 1994). This process of xylem development (xylogenesis) was monitored by sampling wood micro-cores (2 mm in diameter, 1–2 cm in length) from March until mid-October 2008. Sampling was done biweekly in spring and monthly from August onwards in five trees per defoliation class (half of the trees used for dendrochronological analyses) of the two pine species. Samples were taken around the stems at 1.3 m using a Trephor increment puncher (Rossi et al., 2006). The thick dead outer bark was removed, and sampling positions were arranged along an ascending semi-helical pattern in the stem (Deslauriers et al., 2003). The micro-cores were taken about 1 cm apart from each other to avoid wound reaction. The samples usually contained the preceding 4–5 tree rings and the developing annual layer with the cambial zone and adjacent phloem.

Micro-cores were placed in Eppendorf tubes containing a mixed solution of formaldeid, acetic acid and ethanol (5:5:90) and stored as soon as possible at 5°C in order to avoid tissue deterioration. All samples were then processed within a maximum of 1–2 weeks after sampling. Micro-cores were sectioned using a sledge microtome (Anglia Scientific AS 2000, UK) achieving samples 20-μm thick. Sections were mounted on glass slides, stained with 0.5% water solution of cresyl fast violet, fixed with Eukitt® and observed at 100–200x magnification under a light microscope (Olympus BH2).

Four different xylogenesis phases were identified as follows (cf. Wodzicki, 1971; Antonova and Stasova, 1993; Wodzicki, 2001; Deslauriers et al., 2003): (1) cambial cells characterized by small radial diameters, thin walls and bone shape; (2) radially enlarging tracheids presenting unlignified cell walls and therefore unstained in blue; (3) wall-thickening and lignified tracheids with a transition coloration from violet to dark; and (4) mature cells with lignified cells walls fully stained in blue. We counted separately earlywood and latewood mature tracheids and distinguished them by their thin cell walls and wide lumens and thick walls but narrow lumens, respectively. The numbers of cells in each of the four different phases were counted along five radial rows to obtain a mean value per ring and sampling date.

To relate xylogenesis with microclimatic conditions measured *in situ* several climatic variables (air temperature, precipitation, solar radiation, air relative humidity) were recorded hourly and then converted to daily values (mean temperature and radiation, precipitation) using a HOBO microclimate station (Onset, Pocasset, USA) located in each pine stand (see Figure S4).

### Timing of wood formation

Tracheid differentiation was considered to have started and to be complete when at least one horizontal row of cells was detected in the enlarging phase and cell wall thickening and lignification were completed, respectively (cf. Gruber et al., 2010). To precisely define and compare xylogenesis between defoliation classes we computed the onset and cessation dates and the duration of three developmental phases (E, radial enlargement; L, cell-wall thickening and lignification; M, tracheid maturation) using the package CAVIAR in R (Rathgeber et al., 2011). The onset and cessation dates were defined when 50% of the radial files were active (onset) or non-active (cessation) in each xylogenesis phase. The durations of each phase were calculated as the time elapsed between the onset and cessation of these phases following Rathgeber et al. (2011). Xylem formation (X phase) was defined as the time elapsed between the onset of enlargement and the end of maturation. Finally, to compare the onset and cessation dates and the duration of the main phases of xylogenesis we used the achieved significance level (ASL), which can be interpreted in the same way as a *P* significance level since the smaller the value of ASL, the stronger the evidence against a null hypothesis considering no difference between dates or phase durations (Efron and Tibshirani, 1993).

### Statistical analyses

Growth-climate relationships were quantified by calculating Pearson correlation coefficients between daily climate data (mean maximum and minimum temperature, total precipitation, water balance) and ring-width indices. To detect time-dependent growth responses to climate, daily regional climate data were either averaged (temperature) or summed (precipitation, water balance) at 10-day and 15-day long scales following Sánchez-Salguero et al. (2015).

The associations between climate and xylogenesis data (number of cambium cells or tracheids in different developmental stages) were evaluated at 5-, 10-, and 15-day long time scales since daily dynamics of tracheid radial expansion have been described in Scots pine (Antonova et al., 1995). In this analysis we used local climate data recorded in the field during 2008 (mean temperature, precipitation, radiation, relative humidity, water balance). We used linear-mixed effects models to evaluate the effects of defoliation and climate variables on the (*x*^0.5^-transformed) number of different types of tracheids along time, and checked the predicted values and residuals looking for signals of heteroscedasticity (Zuur et al., 2009). Defoliation was regarded as a fixed factor, whereas tree was considered a random factor. Comparison between mean values of tree features (defoliation, size variables) or dendrochronological statistics were based on applying S-N-K *post-hoc* tests. We fitted linear mixed-effects models using the *nlme* library (Pinheiro et al., 2015). All analyses were done using the R statistical program version 3.120 (R Development Core Team, 2015).

## Results

### Post-drought growth patterns

**Figure 1:**
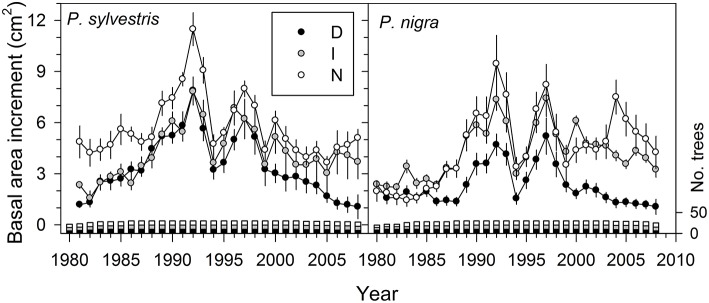
**Recent patterns in basal area increment of the two study pine species according to defoliation intensity (D, severe defoliation, black symbols; I, intermediate defoliation, gray symbols; N, scarce or no defoliation, empty symbols)**. Values are means ± SE (standard errors) and the bars show the annual number of measured trees (right y axis, sample depth) for each defoliation type (same colors are used for growth and sample depth data).

Basal area increment (BAI) dropped in all trees during the dry years 1994–1995, 1999, and 2005 (Figure 1; see also Figure S3). These BAI reductions were followed by a relatively rapid recovery after 1994 and 2005, but not after 1999 in the case of highly defoliated (D) trees of both pine species (Figure 1). We found that BAI for the 2000−2008 period was significantly lower (*P* < 0.05) in the case of D trees (Scots pine, mean ± SE = 2.2 ± 0.4 cm^2^; black pine, 1.6 ± 0.3 cm^2^) as compared with trees presenting intermediate (I) or low (N) defoliation levels whose mean BAI values did not differ (means for I-N trees: Scots pine, 4.3 ± 0.7 cm^2^; black pine, 5.1 ± 0.9 cm^2^). These differences were not associated to tree size which did not differ between defoliation classes (Table 1). Note also that in Scots pine the D and I trees already grew less than the rest of trees in the 1980s and early 1990s, which was not observed in black pine.

Differences in growth between defoliation classes could be traced back in time. Only the D trees showed a lower tree-ring width than the other defoliation classes considering the 1985–2008 period, and this difference was more evident in black pine (Table 2). In Scots pine, the non-defoliated N trees showed the highest first-order autocorrelation in tree-ring width but the lowest mean sensitivity, whereas in black pine the most defoliated D trees presented the highest mean sensitivity (Table 2).

**Table 2:** **Dendrochronological statistics of tree-ring width series for the studied trees and defoliation classes calculated considering the common 1985–2008 period (values are means ± SE)**.

**Species**	**Defoliation class (code)**	**Tree-ring width (mm)**	**First-order autocorrelation^a^**	**Mean sensitivity^a^**
Scots pine (*Pinus sylvestris*)	Severe defoliation (D)	1.93 ± 0.06a	0.75 ± 0.05a	0.38 ± 0.04b
	Intermediate defoliation (I)	2.30 ± 0.12b	0.75 ± 0.08a	0.32 ± 0.03b
	Scarce or no defoliation (N)	2.32 ± 0.07b	0.83 ± 0.02b	0.27 ± 0.04a
*Black pine (Pinus nigra)*	Severe defoliation (D)	1.52 ± 0.05a	0.77 ± 0.05	0.36 ± 0.01b
	Intermediate defoliation (I)	2.20 ± 0.13b	0.75 ± 0.05	0.31 ± 0.02a
	Scarce or no defoliation (N)	2.22 ± 0.14b	0.73 ± 0.05	0.30 ± 0.02a

*Statistics refer to raw data excepting mean sensitivity which was calculated considering residual indices. Different letters show significant differences (P < 0.05) between defoliation classes within each species according to S-N-K post-hoc tests*.

a *The first-order autocorrelation of raw ring-width data measures how much the ring width in year n is correlated with the width in year n-1; the mean sensitivity of residual tree-ring width series measures the relative year-to-year variability in width of consecutive tree rings*.

### Climate-growth relationships

**Figure 2:**
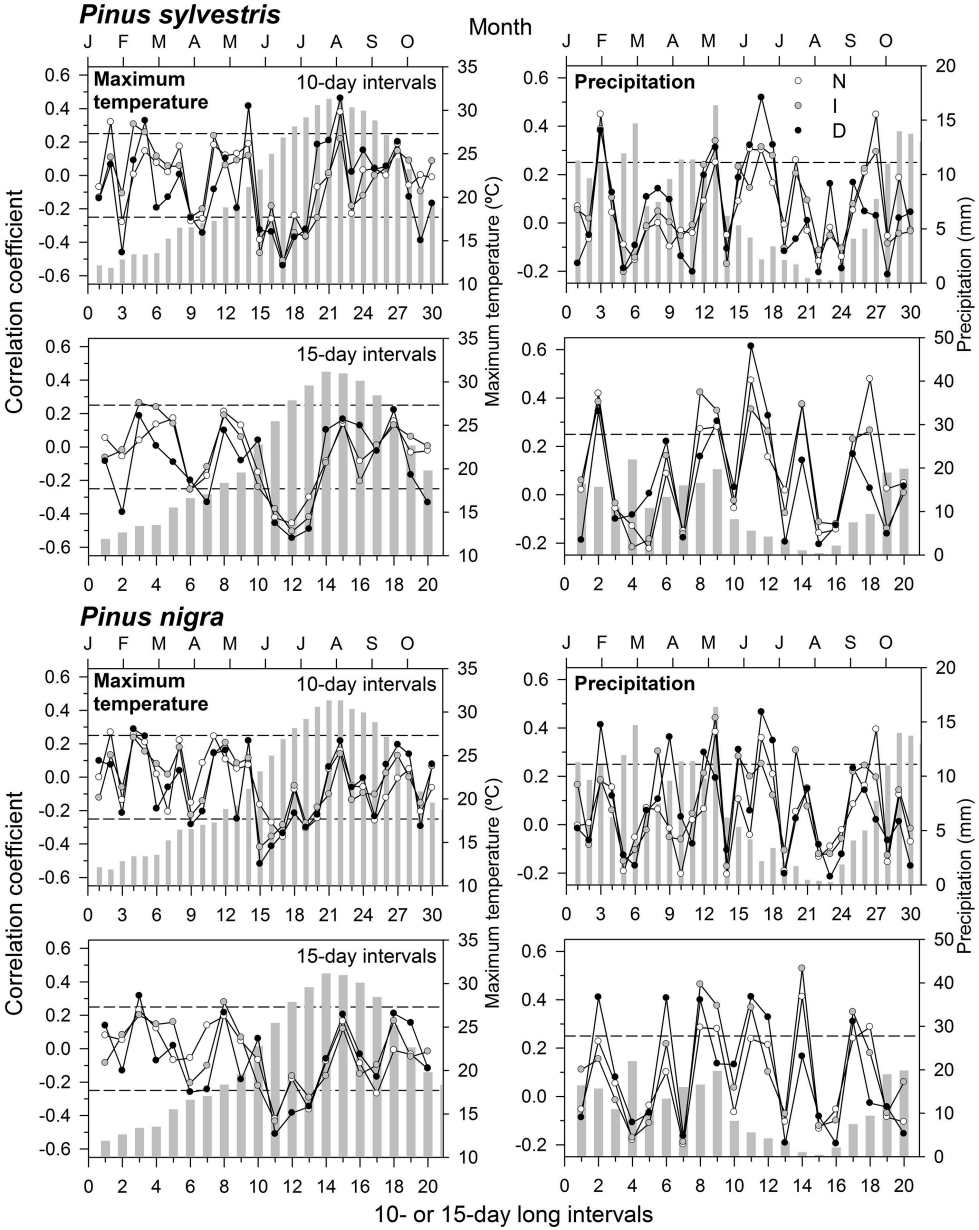
**Climate-growth relationships (Pearson correlation coefficients) calculated for the two pine species and considering three defoliation degrees (N, scarce or no defoliation, empty symbols; I, intermediate defoliation, gray symbols; D, severe defoliation, black symbols)**. Correlations were obtained for 10- and 15-day long intervals considering the 1985–2008 period, and they are presented for mean maximum temperatures and total precipitation. The dashed lines indicate the 0.05 significance levels. The bars show averaged (temperatures) and summed (precipitation) values for each variable.

We found the highest climate-growth correlations when considering mean maximum temperatures and precipitation, which determine water availability during the growing season. Therefore, we present only results for these two climatic variables (Figure 2). In Scots pine, growth was enhanced by wet January and mid-June conditions, with the strongest effect for the latter variable in the case of D trees and at 15-day long intervals (Figure 2). Warm mid-June conditions were associated to low growth in Scots pine, regardless its defoliation level, but high maximum temperatures in early August averaged at 10-day long intervals benefitted growth. In black pine, too warm conditions in early to mid-June were negatively associated to growth of D and I trees, whilst high precipitation values in January and also from May to July were positively associated to growth (Figure 2). In the case of D black pine trees, their growth was most strongly enhanced by June precipitation summed at 10-day long intervals, but a similar response was observed in I trees for July rainfall accumulated at 15-day long intervals.

### Xylogenesis and tree defoliation

**Figure 3:**
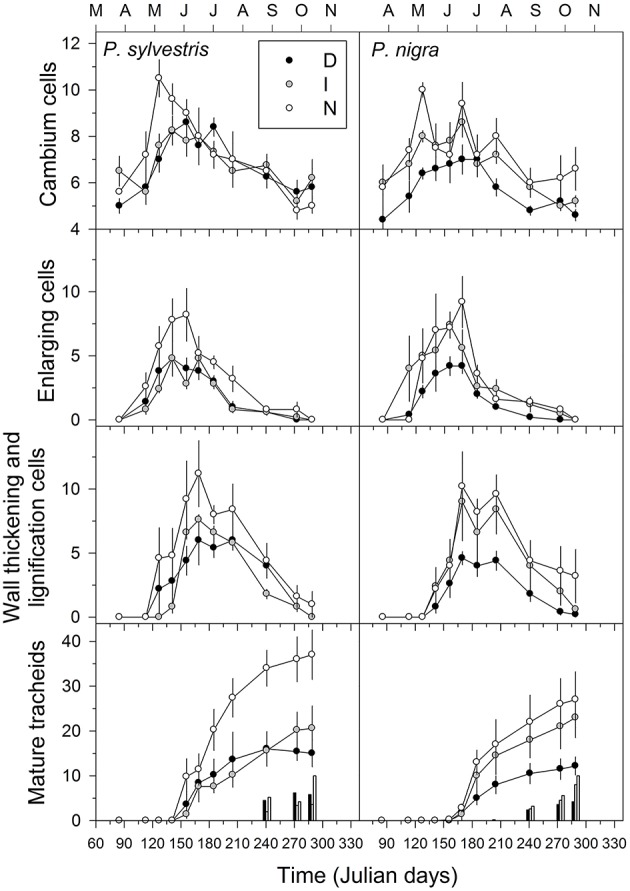
**Number of cambium cells and tracheids observed in different maturation stages (radial enlargement, thickening, maturing) of ***P. sylvestris*** and ***P***. ***nigra*** trees grouped in three defoliation classes (D, severe defoliation; I, intermediate defoliation; N, scarce or no defoliation)**. In the lowermost graph the bars correspond to the number of latewood tracheids. Means are given with standard errors (*n* = 5 trees for each defoliation class).

The D trees produced less tracheids than N trees in the radial enlargement, wall-thickening and lignification, and maturation phases (Figure 3). For instance, on average the D trees produced from 12 (black pine) to 15 (Scots pine) tracheids per tree-ring, whereas the N trees produced 26 (black pine) to 37 (Scots pine) tracheids. These observations were confirmed by the linear mixed-effects models which evidence that defoliation intensity was significantly related to a lower production of radially-enlarging and mature tracheids in both tree species (Table 3). In black and Scots pine, warmer and drier conditions at 15-day long scales were negatively related to the production of radially enlarging tracheids, whereas radiation was positively related to their production (Table 3). However, in Scots pine, the production of cambial cells was positively associated to higher temperatures. Warmer and drier summer conditions enhanced the production of lignifying tracheids.

**Table 3:** **Statistical parameters of the tested factors affecting xylogenesis (number of cambium cells or tracheids in different development stages) in Scots pine (***P. sylvestris***) and black pine (***P. nigra***) trees showing different defoliation levels after drought-induced dieback**.

**Species**	**Effects**	**No. cambial cells**			**No. radially-enlarging tracheids**			**No. wall-thickening tracheids**			**No. mature tracheids**		
		**5 days**	**10 days**	**15 days**	**5 days**	**10 days**	**15 days**	**5 days**	**10 days**	**15 days**	**5 days**	**10 days**	**15 days**
Scots pine (*Pinus sylvestris*)	Tree		0.12			0.22			0.28			0.09	
	Defoliation		−0.17			−**0.57**			−0.14			−**2.17**	
	Time	0.02	0.01	0.03	−**0.29**	−**0.28**	−**0.34**	0.01	0.06	0.07	**0.21**	**0.28**	**0.26**
	Mean temperature	0.04	0.31	**0.63**	**0.58**	−0.26	−**0.89**	0.11	**0.24**	**0.44**	0.19	0.16	0.15
	Relative humidity	0.06	0.01	0.05	**0.28**	0.06	−0.01	**0.23**	0.18	0.20	0.18	0.15	0.14
	Radiation	0.04	**0.21**	**0.33**	−0.01	**0.44**	**0.48**	−0.03	0.16	0.18	−0.16	−0.13	−0.09
	Precipitation (P)	0.04	**0.08**	**0.12**	0.05	**0.22**	**0.28**	0.01	0.06	0.05	0.10	0.14	0.10
	Water balance	−0.05	−0.05	−**0.11**	−0.04	−**0.20**	−**0.27**	−**0.24**	−0.07	−0.03	−0.08	−0.20	−0.09
	$R_{\text{adj}}^{2}$ (%)	29.61	28.54	33.60	38.97	39.60	45.95	44.51	41.20	45.62	34.45	33.89	34.14
Black pine (*Pinus nigra*)	Tree		0.03			0.17			0.03			0.06	
	Defoliation		−**0.55**			−**0.78**			−**1.15**			−**5.16**	
	Time	0.04	0.02	0.02	−**0.24**	−**0.25**	−**0.27**	0.06	**0.20**	**0.21**	**0.22**	**0.23**	**0.27**
	Mean temperature	0.07	0.06	0.02	**0.58**	−0.04	−**0.94**	0.04	**0.72**	**0.84**	0.14	0.12	0.10
	Relative humidity	0.03	0.05	0.01	**0.24**	0.04	0.06	0.01	0.03	0.04	0.12	0.04	0.11
	Radiation	0.04	**0.20**	**0.32**	−0.01	**0.52**	**0.53**	0.05	0.01	0.03	−0.10	−0.08	−0.03
	Precipitation (P)	0.01	0.01	0.03	0.01	**0.20**	**0.27**	0.04	0.08	0.04	0.03	0.07	0.20
	Water balance	−0.01	−0.04	−0.02	−0.05	−**0.19**	−**0.22**	−**0.15**	−**0.22**	−**0.29**	−0.05	−0.08	−0.14
	$R_{\text{adj}}^{2}$ (%)	29.81	28.70	32.24	41.26	37.45	46.38	38.70	42.76	48.33	46.82	45.72	45.99

*The last line of each model gives the adjusted R^2^ ($R_{\mathrm{adj}}^{2}$), which measures the proportion of variance of the dependent variable explained by the model. All models were evaluated for 5-, 10-, and 15-day long intervals. Bold values indicate significant effects (P < 0.05)*.

**Figure 4:**
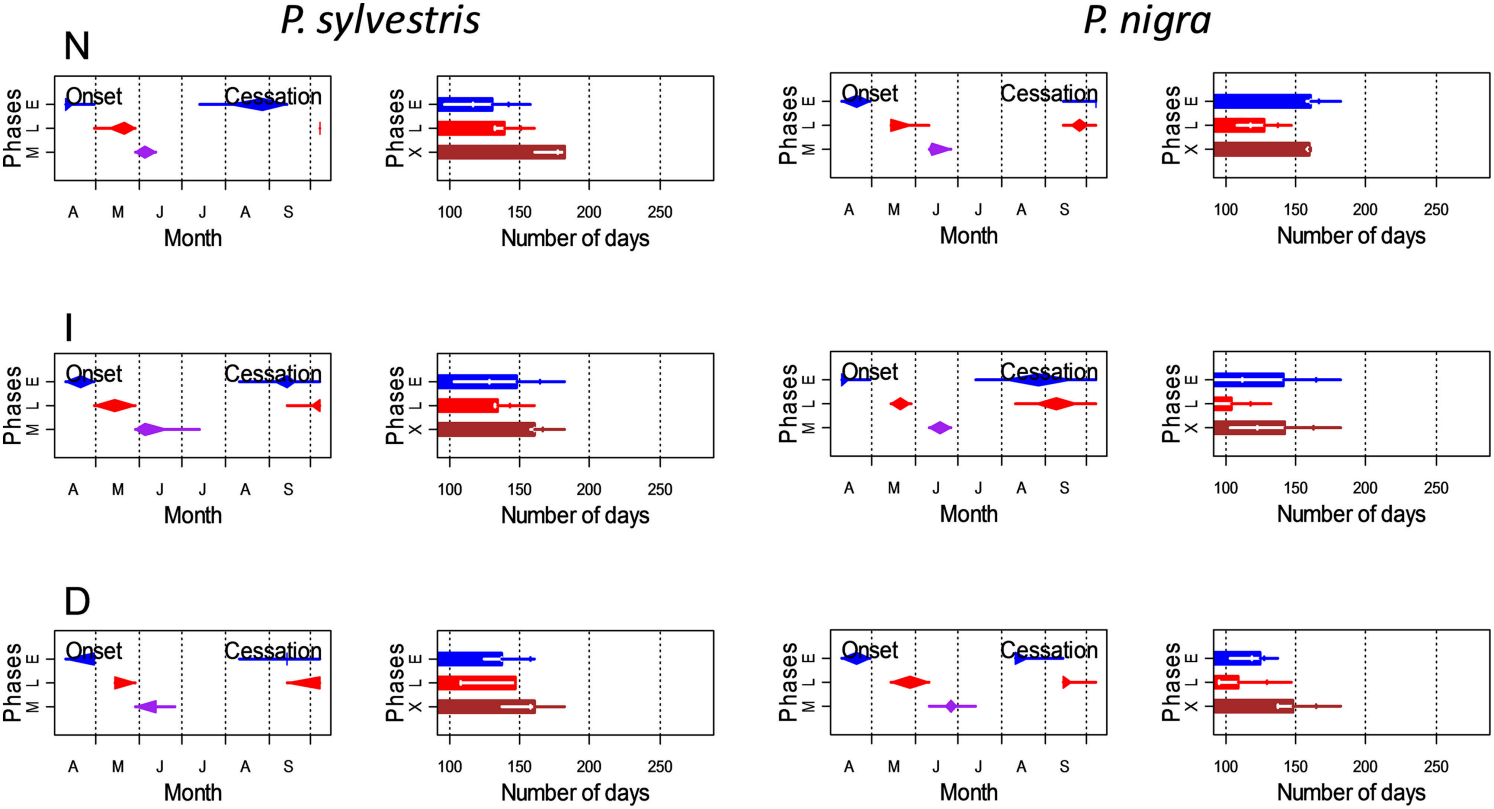
**Estimated onset and cessation dates and durations (±SD) of the main phases of xylogenesis (E, radial enlargement; L, lignification and cell-wall thickening; M, mature tracheids; X, xylem formation) in Scots pine (***P. sylvestris***) and black pine (***P. nigra***) trees of different defoliation classes (D, severe defoliation; I, intermediate defoliation; N, scarce or no defoliation)**. Onset and cessation dates of selected xylogenesis phases are represented by diamond-crossed-by-a-line marks whose left (right) end of the line represents the minimum (maximum), the left (right) end of the diamond the first (third) quartile and the middle of the diamond corresponds to the median.

In both pine species the number of cambial cells of N trees reached the highest value in May, but peaked 1 month later in the case of D trees (Figure 3). This means that the onset of xylem formation started earlier in the N than in the D trees. In the case of the radial-enlargement phase, the onset occurred significantly earlier in N than in D trees (Table 4). The peak of formation of radially-enlarging tracheids occurred from May to June in Scots pine and around mid-June in black pine. This phase ended before in the D trees than in the other defoliation classes in both pine species (Table 4, Figure 4). Consequently, the D trees were characterized by presenting the shortest duration of the radial-enlargement phase, but this difference was only significant in black pine (D, 128 days vs. N trees, 160 days; Table 4). The wall-thickening and lignification phase was similar among defoliation classes showing a maximum activity from June to July in both pine species. Lastly, maturation proceeded similarly in the three defoliation classes, albeit latewood formation seemed to start earlier in the case of Scots pine D trees but we could not assess if there were significant differences. Overall, the duration of xylem formation was shorter in D trees than in the other types of trees.

**Table 4:** **Statistical tests (ASL) obtained by comparing the estimated onset and cessation dates and the duration of the main phases of tracheid differentiation for ***P. sylvestris*** and ***P. nigra*** trees of different defoliation classes (D, severely defoliated; I, intermediate defoliation; N, scarcely or not defoliated)**.

**Species**	**Event**	**Xylogenesis phase**	**D vs. N**	**D vs. I**	**I vs. N**
Scots pine (*Pinus sylvestris*)	Onset	Radial enlargement	**0.012**	0.136	0.076
		Cell-wall thickening	0.059	0.362	0.081
	Cessation	Radial enlargement	**0.021**	**0.042**	**0.047**
		Cell-wall thickening	0.069	0.188	0.073
	Duration	Radial enlargement	0.075	0.251	0.212
		Cell-wall thickening	0.061	0.080	0.287
		Xylem formation	**0.026**	0.333	0.109
Black pine (*Pinus nigra*)	Onset	Radial enlargement	0.078	0.091	0.080
		Cell-wall thickening	0.084	0.054	0.069
	Cessation	Radial enlargement	**0.019**	**0.037**	**0.025**
		Cell-wall thickening	0.065	0.056	0.077
	Duration	Radial enlargement	**0.032**	0.190	0.154
		Cell-wall thickening	0.237	0.066	0.187
		Xylem formation	**0.048**	0.190	0.084

*Significant (P < 0.05) ASL values, based on 10,000 bootstrapped iterations, appear in bold characters. Note that the smaller the value of ASL, the stronger the evidence to support a significant difference*.

## Discussion

Here we document how drought-induced dieback caused growth decline and affected xylogenesis in the widely distributed Scots pine and the Circum-Mediterranean black pine. The duration of xylem formation was shorter in the defoliated than in the non-defoliated or moderately defoliated trees in both species (Table 4), which agrees with the fact that the most defoliated trees grew less and thus produced less tracheids than the other types of trees (Figures 1, 3). Defoliated pines form narrow tree rings as the result of a shorter growing season due to a later onset of xylogenesis or a premature cessation of cambial activity and wood formation as has been observed by others (Bauch et al., 1979; Eilmann et al., 2011, 2013). Note that the duration of xylogenesis phases as the radial enlargement of tracheids, which depends on an adequate turgor pressure of expanding cells (Abe et al., 2003), drives to a great extent the annual ring width and the final size of tracheids (Cuny et al., 2014). However, we could only find a significantly shorter duration of the radial-enlargement phase in the most defoliated black pine trees as compared with their less defoliated conspecifics (Table 4).

The long-term climate-growth associations showed a pronounced sensitivity of growth in defoliated Scots pine trees to changes in precipitation during the growing season (Figure 2). This agrees with the fact that Scots pine is more vulnerable to drought-induced xylem embolism than black pine (Martínez-Vilalta et al., 2004). This species was also the most negatively affected by the 1990s and 2000s droughts in the study area (Sánchez-Salguero et al., 2012a). Such sensitivity to water availability also agrees with the highest year-to-year variability in growth presented by the most defoliated Scots pine trees (Table 2). From this point of view, Scots pine could be considered less adapted to global-change-type droughts than black pine. However, climate-xylogenesis associations did not indicate greater drought sensitivity in Scots pine as compared with black pine (Table 3). Scots pine responds to drought by a fast reduction of transpiration through a rapid stomatal closure (Irvine et al., 1998), but this response varies between trees as a function of their stress level (Hölttä et al., 2012). Such isohydric behavior combined with needle shedding could compensate the alterations in source-sink relationships within the tree (Iqbal et al., 2012). However, growth data indicate that growth decline is irreversible in this species, and also in black pine, for very high defoliation levels and after three severe droughts as those which occurred in 1994–1995, 1999, and 2005 (Figure 1). Overall, our findings suggest that defoliated trees regulated their water status after the severe 1990s and 2000s droughts by needle shedding so as to keep a stable ratio between conductive area and transpiring area. The most defoliated trees presented the lowest growth rates prior to the droughts but we do not know if they were those transpiring most actively and therefore losing more water through their stomata. Whatever the cause, such low-growth trees were the most prone to drought-induced alterations in their hydraulic system, defoliation, changes in xylogenesis, and reduced growth after the drought. Drought-induced severe defoliation possibly portends tree death in the most affected trees.

The association between defoliation and a reduced growth rate was observed for all the assessed xylogenesis phases in black pine, and for the radially-enlarging and mature tracheids in Scots pine (Figure 3, Table 3). This is consistent with the finding that the most defoliated black pine trees were characterized by presenting the shortest duration of the radial-enlargement phase, a stage which is tightly related to the growth rate of trees (Horacek et al., 1999). A reduced number of enlarging cells was also found when imposing water deficit on black spruce (*Picea mariana*) saplings under controlled conditions (Balducci et al., 2013). Notably, the ratio between the numbers of total mature tracheids in the non-defoliated as compared with the most defoliated trees was similar between the two species (ca. 2.5). This could indicate that drought-triggered defoliation caused a similar growth reduction in both species despite the aforementioned differences regarding xylogenesis.

It is also remarkable that observational (Torelli et al., 1986) and empirical (Balducci et al., 2016) studies did not detect clear modifications of transversal tracheid dimensions in silver fir (*Abies alba*) trees showing dieback or in black spruce saplings experiencing imposed water deficit, respectively. However, the stem wood of defoliated silver fir trees showed a higher susceptibility to decay implying different lignifications processes (Shortle and Ostropsky, 1983). In fact, silver fir trees showing dieback produced narrow rings due to a premature end of wood formation characterized by an earlier differentiation of the latewood cell walls as compared with non-declining trees (Torelli et al., 1999). We could not find significant differences between defoliation classes regarding the cell-wall thickening and lignification phase, despite there was a trend toward an earlier cessation of this phase in the most defoliated trees, particularly in the case of black pine. These results suggest that xylogenesis is more sensitive or plastic to water shortage and defoliation than wood anatomy. However, both responses are not mutually exclusive. Dry conditions can stop cambial activity, shorten the growing period, and also induce the formation of tracheids with wider conduits and thinner cell walls as has been described in Scots pine adult trees (Eilmann et al., 2011).

The plasticity of xylogenesis in response to seasonal or punctual water shortage has been profusely documented in empirical and observational studies. In a warming and drought experiment considering black spruce saplings, water shortage reduced the rates of cell production (Balducci et al., 2016), as previously observed in experiments with Aleppo pine saplings (De Luis et al., 2011). Field studies also reported plastic responses of cambial activity to seasonal water availability (e.g., bimodal behavior) in either Mediterranean species as Aleppo pine (De Luis et al., 2007; Camarero et al., 2010) or Eurosiberian species as Scots pine growing at xeric sites in the Alps (Gruber et al., 2010; Eilmann et al., 2011; Oberhuber et al., 2011). In a rainfall exclusion experiment applied to Scots pine, the radial-enlargement phase was shortened in trees subjected to drier conditions compared with control trees although this difference was not significant and depended on tree size (Fernández-De-Uña et al., 2013). Overall, these studies indicate that cambial activity is greatly reduced by drought but can rapidly resume once water availability increases (Eilmann et al., 2011, 2013).

Plastic xylogenesis could represent a strategy to respond to changes in water availability and the reduction of photosynthetic area through leaf shedding. In a Scots pine forest located at a xeric site in the Swiss Alps and subjected to a drought trees with medium to high defoliation grew less and showed a shorter growth period than non-defoliated trees (Eilmann et al., 2013), which fully agrees with our findings. When irrigation occurred in this site all trees responded positively and rapidly showing enhanced radial growth and stopping needle shedding, irrespective of their defoliation degree, which is in accordance with previous studies (Dobbertin, 2005). The reduction of the production rates of tracheids in different xylogenesis phases of the most defoliated trees, particularly when cells are radially enlarging, was not compensated by longer durations of these phases as was suggested in a drought experiment (Balducci et al., 2016). Following this line of reasoning, it has been suggested that drought and subsequent defoliation characterizing dieback episodes could lead to the depletion of carbon stores (Galiano et al., 2011). However, other authors indicate that water shortage is a more relevant and direct constrain of growth than a reduced availability of non-structural carbohydrates (Sala et al., 2012). In fact, defoliation caused by insects such as the pine processionary moth (*Thaumetopoea pityocampa*), which particularly affects black pine, reduce radial growth but not the concentrations of non-structural carbohydrates (Palacio et al., 2012; Puri et al., 2015). Seasonal changes in sugar concentration within the cambial zone have been linked to xylogenesis and peak when most radial growth is finished, i.e., during the wall-thickening and lignifications phase (Simard et al., 2013). Nonetheless, this does not mean that cambial activity of trees is directly limited by the availability of carbohydrates because drought can lead to the use of soluble sugars for osmoregulation but also reduce cell turgor, expansion, and lignification as well as related cambial dynamics (Deslauriers et al., 2014).

We found the reported differences in xylogenesis in the most defoliated trees 3 years after the severe 2005 drought induced dieback and triggered needle loss (Sánchez-Salguero et al., 2012b). This implies that once crown defoliation reaches a threshold (in this case above 75%) the ability of trees to recover growth could be compromised in some pine species or at xeric sites if water availability improves. Globally, legacy effects of droughts cause lags of 2–4 years for the recovery of forest growth (Anderegg et al., 2015). We show that defoliation could lengthen these recovery periods and compromise the resilience of forests experiencing drought-induced dieback. Lastly, we extract a practical lesson of this study. Xylogenesis studies are highly time consuming. Nevertheless, to reach more general and robust conclusions we suggest sampling 10 trees per defoliation class in further studies. Sampling could be done weekly during the most active growing period (for instance from April to July in our case) and biweekly the rest of the year.

To conclude, drought negatively impacted growth and crown cover in Scots pine and black pine. In the most defoliated trees, the duration of xylem formation and the radial-enlargement phase shortened leading to low growth rates and the formation of narrow rings. In Scots pine, the onset of xylem formation was retarded in the most defoliated trees as compared to non-defoliated trees. Despite the widely reported plastic responses of cambial activity to changing water availability, we found a very limited resilience capacity of Scots and black pines after drought in severely (≥75%) defoliated trees. Moreover, droughts produce legacy effects on xylogenesis of these severely defoliated trees which show irreversible growth decline and are prone to die.

## Author contributions

GG and RS performed the data collection in the field and contributed significantly to data analysis, discussing the results, and writing of the paper. JC conducted the statistical analysis and the manuscript redaction. RN has contributed to the study design and the manuscript discussion and approval.

### Conflict of interest statement

The authors declare that the research was conducted in the absence of any commercial or financial relationships that could be construed as a potential conflict of interest.
