# Supplementary material for: Limited Growth Recovery after Drought-Induced Forest Dieback in Very Defoliated Trees of Two Pine Species
Source: Front Plant Sci. 2016 Apr 1;7:418. doi: 10.3389/fpls.2016.00418 (PMC4817349; doi:10.3389/fpls.2016.00418)
Supplement: Supplementary file 1 [file DataSheet1.DOCX]

***Supplementary Material***

**Limited growth recovery after drought-induced forest dieback in very defoliated trees of two pine species**

**Guillermo Guada^1^, J. Julio Camarero^2^*, Raúl Sánchez-Salguero^3^, Rafael M. Navarro Cerrillo^4^**

***Correspondence:** Dr. J. Julio Camarero: jjcamarero@ipe.csic.es

**1. Supplementary Figures and Tables**

Supplementary material includes four figures and one table. The table provides details on the meteorological records used to calculate the regional averages. The figures show the location of the study, a climate diagram, trends observed in seasonal precipitation, monthly values of the Standardised Precipitation-Evapotranspiration Index (SPEI) and main climatic variables recorded at the Scots pine study site.

## 1.1. Supplementary Figures


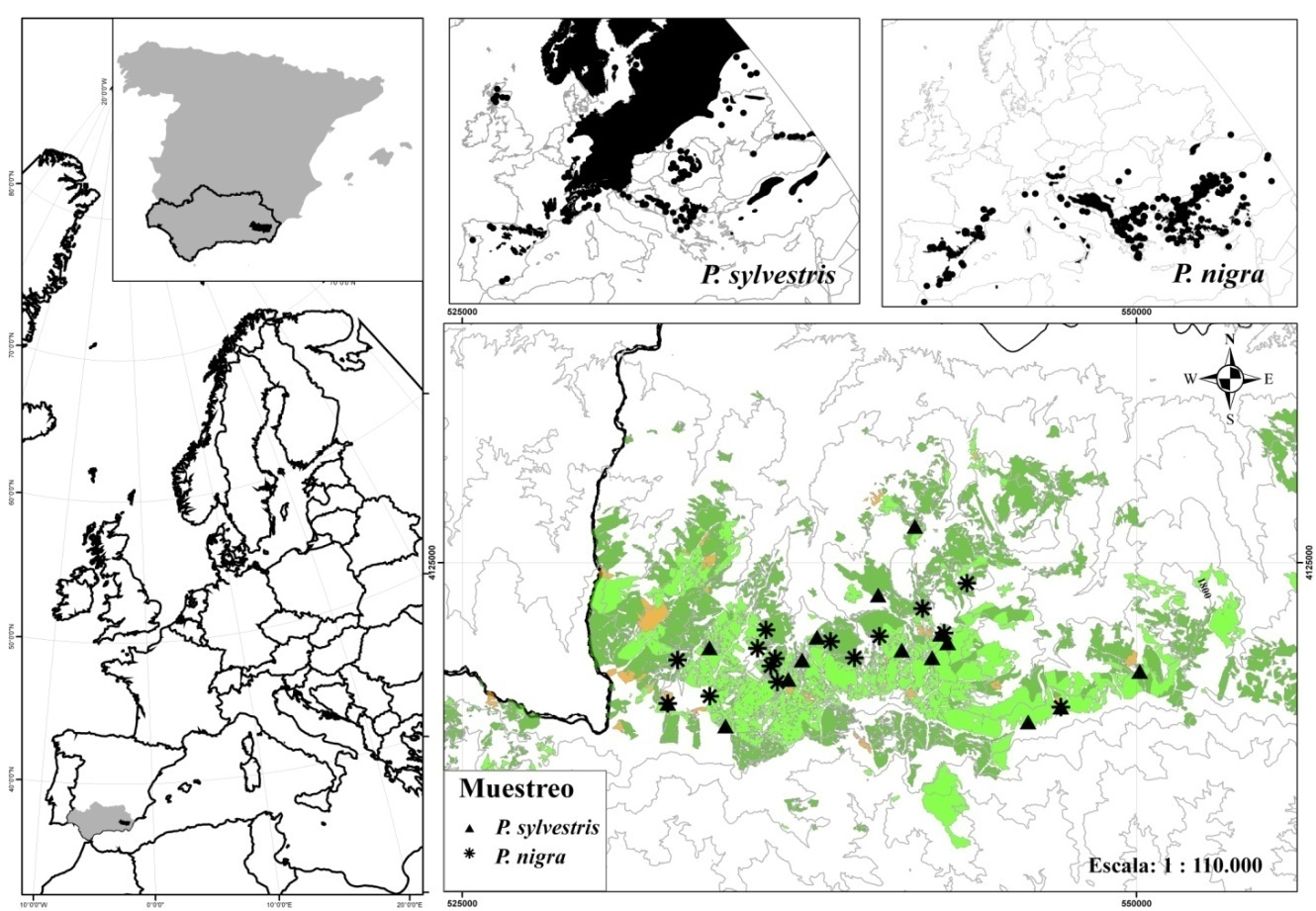


Trees

10 km

**Supplementary Figure S1**. Location of the study area in “Sierra de Filabres” (Andalusia, SE Spain) as compared with the distribution area of the two study pine species, the widely distributed Eurasian Scots pine (*P. sylvestris*) and the Mediterranean black pine (*P. nigra*). The lower right map shows the location of sampled trees (*P. sylvestris*, triangles; *P. nigra*, asterisks) and the area covered by each species in the “Sierra de Filabres” range (*P. sylvestris*, clear green polygons; *P. nigra*, dark green polygons; mixed stands, orange polygons).

**Supplementary Figure S2.** Climate diagram based on the regional climatic series calculated for the Sierra de Filabres study area, SE Spain.

1.

(a)

(b)

**Supplementary Figure S3.** Trends observed in seasonal precipitation (a, regional data) and drought severity (b) at Sierra de Filabres study sites (SE Spain). The statistics of rainfall data show the trends calculated using the non-parametric tau (τ) coefficient and its associated probability level (*P*). Vertical dashed lines indicate dry years (1994-1995, 1999 and 2005) corresponding to sharp growth declines. The lowermost plot (b) shows the monthly values of the Standardised Precipitation-Evapotranspiration Index (SPEI) for the same period, calculated for 3-, 6 and 12-month long scales since these scales are the most important for the study pine species (Pasho *et al*., 2012). The lines show loess-smoothed SPEI values with negative and positive SPEI values indicating dry and wet conditions, respectively. Note that the 2005 drought was characterized by very negative SPEI values at the 6-month long scale and during the second half of the year. The SPEI was calculated for the 0.5º grid including both study sites and it was obtained from the webpage <http://sac.csic.es/spei/index.html> (accessed April 20 2015).

**Supplementary Figure S4.** Main climatic variables measured at the Scots pine study site in Sierra de Filabres (SE Spain) during the year 2008 when xylogenesis was characterized. Note that negative air temperatures occurred from January to mid April, and also from late October to December.

**1.2** **Supplementary Tables**

**Table S1.** Meteorological stations used to calculate the regional mean series of the study area for the 1970-2008 period.

| Station | UTMX (N) | UTMY (W) | Altitude (m) | Precipitation record | Annual precipitation (mm) | Missing values (%) | Temperature record | Mean annual temperature (ºC) | Missing values (%) |
| --- | --- | --- | --- | --- | --- | --- | --- | --- | --- |
| Serón | 543465 | 4132891 | 850 | 1970-1999 | 324 | −−− | 1970-1999 | 14.7 | −−− |
| Alcontar | 536913 | 4132000 | 954 | 1961-2008 | 308 | 10.4 | −−− | −−− | −−− |
| Hueneja | 505919 | 4113431 | 1166 | 1940-1999 | 432 | −−− | 1940-1999 | 12.8 | −−− |
| Bacares | 548779 | 4122816 | 1200 | 1901-2008 | 344 | −−− | 1950-2008 | 12.7 | −−− |
| Los Santos | 535475 | 4122749 | 1600 | 1961-2008 | 382 | 4.0 | −−− | −−− | −−− |
| Calar Alto | 539927 | 4119071 | 2168 | 2001-2008 | 297 | 5.1 | 1997-2008 | 6.9 | 4.8 |
